# Clinicopathological features, risk model and prognosis of 115 cases of epithelioid hemangioendothelioma: A single-center study

**DOI:** 10.3389/fonc.2025.1577968

**Published:** 2025-08-21

**Authors:** Shihui Zhang, Yiting Liang, Ye Yang, Lei Guo, Weihua Li, Susheng Shi

**Affiliations:** Department of Pathology, National Cancer Center/National Clinical Research Center for Cancer/Cancer Hospital, Chinese Academy of Medical Sciences and Peking Union Medical College, Beijing, China

**Keywords:** epithelioid hemangioendothelioma, CAMTA1, TFE3, prognosis, risk model

## Abstract

**Objective:**

To investigate the clinicopathological features, diagnostic value, risk model and prognostic significance of epithelioid hemangioendothelioma (EHE) in a retrospective cohort of 115 cases.

**Methods:**

A total of 115 cases of EHE diagnosed in the Cancer Hospital of the Chinese Academy of Medical Sciences (NCC) from 2011 to 2023 were collected. The clinical and pathological features of EHE were reviewed by Fluorescence *in situ* hybridization (FISH) and Immunohistochemistry (IHC). SPSS 25.0 software for the overall survival (OS) curve. Univariate and multivariate COX proportional risk regression models were used to analyze the prognostic factors.

**Results:**

The male to female ratio of 115 patients was 1.05:1. The age of the patients ranged from 8 to 84 years (median, 47 years; standard deviation (SD), 15.055), and tumor diameter ranged from 8 to 152 mm (median, 20 mm; SD, 29.156).Among them, there were 80 multiple cases, 69 cases of the patients underwent surgery. IHC showed that 92.2% of calmodulin-binding transcription activator 1 (CAMTA1) and 58.0% of transcription factor E3 (TFE3) were positively expressed. The positive rate of the WWTR1::CAMTA1 fusion gene was 86.7% and the positive rate of the TFE3 fracture gene was 13.6% (12/88). The difference test between FISH and IHC showed that the two detection methods have good consistency for CAMTA1 gene detection, while the consistency with TFE3 is poor. Univariate COX regression showed that radical surgical resection, tumor size (>5cm) and age (>50 years), multi-organ involvement, and OS were statistically significant (P<0.05). A proposed 3-tiered risk assessment system using these 5 parameters significantly stratified the patients into low-risk, intermediate-risk and high-risk groups with significantly OS rates.

**Conclusion:**

The prognosis for EHE patients with tumor size more than 50 mm or age at diagnosis over 50 years old is unfavorable. In this investigation, we pioneered the development of a prognostic risk model, leveraging five key parameters to anticipate the outcomes for EHE patients.

## Introduction

Epithelioid hemangioendothelioma (EHE), a vascular tumor of low to intermediate malignancy potential, exhibits distinct epithelioid characteristics and predominantly affects patients aged 40 to 60 years. It is typically indolent and most commonly involves the liver, lungs, and bone. Histologically, EHE is characterized by epithelioid tumor cells arranged in nests within a hyalinized stroma, featuring cytoplasmic eosinophilia and the presence of intracytoplasmic lumina.

The most prevalent molecular aberration encountered in EHE patients is the translocation t(1;3)(p36.3;q25), which triggers the fusion of WWTR1, a transcriptional regulator containing a WW domain located on 3q25, with CAMTA1 located on 1p36.3 (1). Additionally, a subset of cases harbors a t(X;11)(p11;q22) translocation, leading to the fusion between YES-associated protein 1 (YAP) and TFE3 (2). While research into EHE has intensified in recent years, generating numerous reports detailing the biological characteristics and histological presentations of EHE, comprehensive clinicopathological data and survival outcomes specifically for Chinese EHE patients remain scarce. To address this gap, we conducted a comprehensive review of 115 EHE cases from multiple anatomical sites, focusing on histological features and molecular characterization. We further evaluated the diagnostic utility of Fluorescence *In Situ* Hybridization (FISH) and Immunohistochemistry (IHC) in EHE. Additionally, we investigated the impact of clinical factors on EHE prognosis, aiming to enhance our understanding of the disease and the value of different diagnostic tests in its management.

## Materials and methods

### Study populations and follow-up procedure

This retrospective observational study was conducted at a single institution, encompassing NCC cohort of 115 patients diagnosed with Epithelioid Hemangioendothelioma (EHE) between January 1, 2014, and December 31, 2023. The clinicopathological variables of all patients, including sex, age at time of diagnosis, maximum tumor size and so on were evaluated comprehensively. The inclusion criteria comprised patients diagnosed with EHE at NCC between 2011 and 2023 who had complete clinicopathological information and follow-up records. The exclusion criteria were: patients who did not undergo surgery at NCC or whose postoperative specimens were unavailable; patients lacking complete clinicopathological information; and those lost to follow-up.

It is noteworthy that some cases were consultation referrals from other hospitals, resulting in limited availability of Immunohistochemical (IHC) staining data for all markers in all cases. The study was conducted in accordance with ethical guidelines and approved by the Ethics Committee of the National Cancer Center/Cancer Hospital, Chinese Academy of Medical Sciences. Given its retrospective nature, data analysis was conducted anonymously, waiving the requirement for informed consent. OS was defined as the interval from the date of surgery until death or the most recent follow-up.

### IHC

All specimens underwent fixation using 10% neutral formalin. For histological examination, wax blocks enriched with tumor tissue and adjacent normal tissue were meticulously selected and subjected to serial sectioning at a thickness of 4 microns. These sections were then stained with Hematoxylin and Eosin (HE) and examined under light microscopy. Immunohistochemical (IHC) staining was performed employing the EnVision two-step method. Monoclonal antibodies against ERG (1:100, clone MXR004, MAXIM, China), CD34 (1:100, Q8End/10, MAXIM, China), CD31 (1:100, MX032, MAXIM, China), and TFE3 (1:100, MRQ-37, MAXIM, China) were incubated overnight at 4°C. Meanwhile, polyclonal antibodies against FVIII (1:400, MAXIM, China) and CAMTA1 (1:200, Abcam, USA) were also incubated under the same conditions.

For IHC interpretation, we adopted a semi-quantitative approach. Positive expression was indicated by the presence of a brown-yellow coloration in either the nucleus or cytoplasm. Specifically, positive staining for CD34, CD31, FVIII, and CAMTA1 was localized within the cytoplasm, whereas positive staining for ERG and TFE3 was observed in the nucleus. A tumor was considered IHC-positive if more than 5% of the tumor cells exhibited staining, and negative if there was no staining or staining in ≤5% of the tumor cells. All IHC staining sections were interpreted by 2 pathologists in a blinded manner.

### FISH

FISH analysis was conducted utilizing specific probe reagents for the detection of WWTR1::CAMTA1 fusion gene t(1;3) (Accupath, China), TFE3 gene break-apart at Xp11.2 (Accupath, China), and YAP1 gene break-apart at 11q22 (Accupath, China). A positive result was defined by the presence of cells showing either a fusion signal (red/green co-localization) or separated red and green signals (indicative of gene breakage). For each case, 100 tumor cell nuclei were evaluated. Samples were considered positive if >15% of cells were positive, and negative if <15% were positive. For cases with 5-15% positive cells, an additional 100 tumor cells were counted to reach a definitive conclusion.

### Statistical analysis

All statistical analyses and figure creations were meticulously conducted using SPSS version 25.0 (IBM, Armonk, NY, USA) and R Studio version 4.3.2 (Boston, Massachusetts, USA). Descriptive statistics were comprehensively presented, encompassing percentages, medians, and ranges to provide a comprehensive overview of the data. To evaluate the associations between the FISH test and IHC test, Chi-square tests and Kappa tests were employed. For the comparison of survival rates, the Kaplan-Meier method with a log-rank test was utilized, offering insights into the differences in survival outcomes. Furthermore, hazard ratios were derived through both univariate and multivariate Cox regression models, facilitating a deeper understanding of the factors influencing survival. All confidence intervals (CIs) were reported at the 95% confidence level, ensuring the robustness of our findings. A P-value of less than 0.05 was deemed statistically significant, indicating a high level of certainty in our conclusions.

## Results

### Clinicopathologic features in EHE

The median age at diagnosis was 47 years (range: 8–84 years), with females comprising 48.7% (56/115) of the cohort. Regarding lesion distribution, 30.4% (35/115) of patients presented with a single lesion, while 69.6% (80/115) had multiple lesions. Tumor involvement was confined to a single organ in 88.7% (101/115) of patients; 11.3% (14/115) exhibited multi-organ involvement. The liver was the most common tumor site (46.1%, 53/115), followed by the lungs (29.6%, 34/115), bone and skin (21.7%, 25/115), and other sites (6.1%, 7/115). The median tumor size was 20 mm (range: 8–152 mm).Treatment modalities included surgery (radical resection or TACE) in 60.0% (69/115) of patients and conservative management (radiation, chemotherapy, or palliative care) in 40.0% (46/115).The median follow-up duration was 912 days (range: 11–4882 days). During follow-up, 18.3% (21/115) of patients died (**Table 1**).

**Table 1:** Baseline clinicopathologic feature in EHE.

Clinicopathological parameters	N (%)
Gender	
Male	59 (51.3)
Female	56 (48.7)
Age (years)	
Median (range)	47 (8-84)
Maximum tumor size (mm)	
Median (range)	20 (8-152)
Pathogenic site	
Liver	53 (46.1)
Lung	34 (29.6)
Skin and bone	21 (21.7)
Others	7 (6.1)
Treatment modalities	
Surgery	65 (60.0)
conservative treatment	50 (40.0)
Single/multiple lesions	
Single lesions	35 (30.4)
Multiple lesions	80 (69.6)
multi-organ/single-organ tumors	
multi-organ tumors	14 (11.3)
single-organ tumors	101 (88.7)
Survival status	
Dead	21 (18.3)
Alive	94 (81.7)

### Different detection methods of EHE

**Figure 1:**
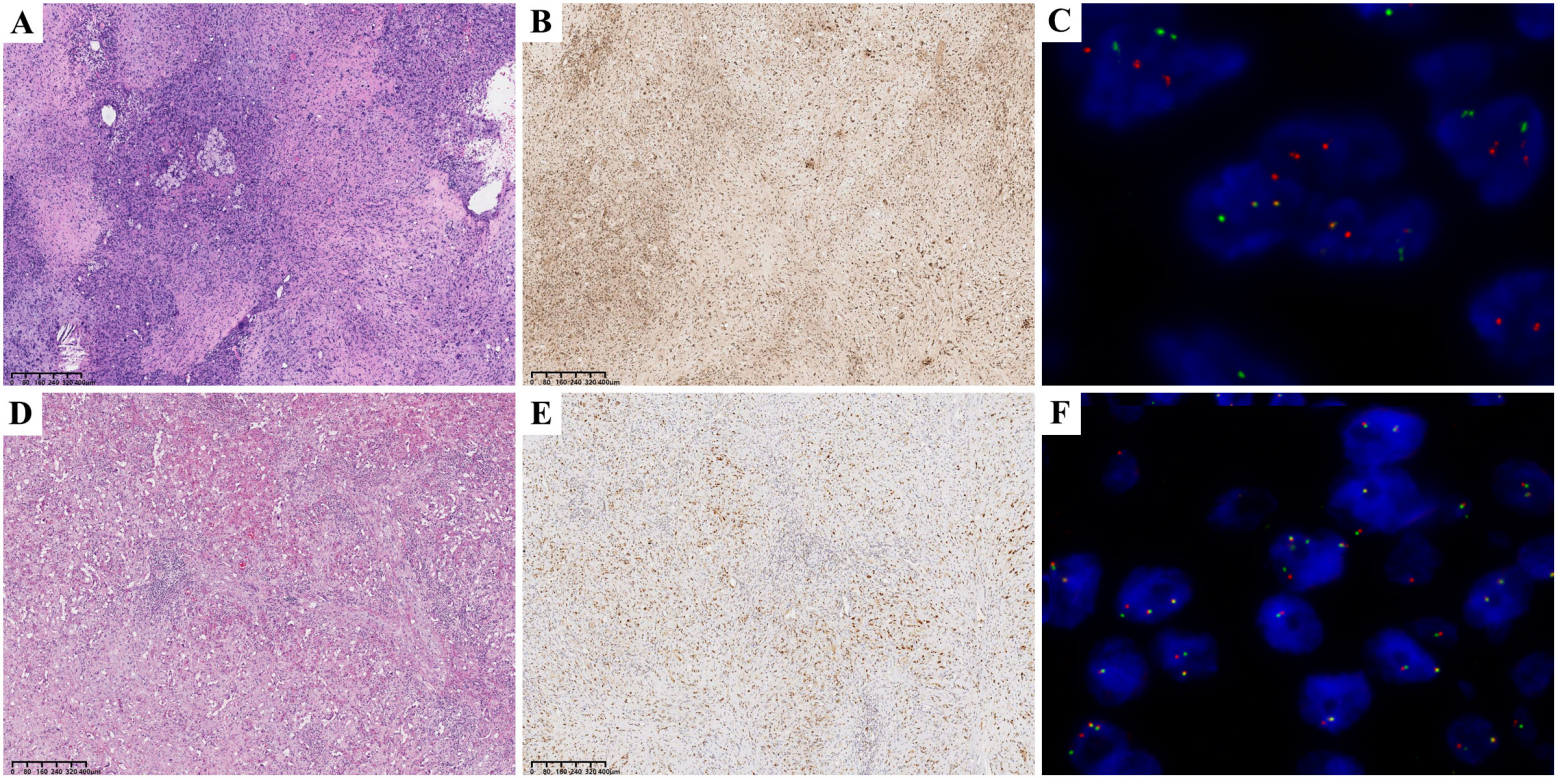
CAMTA1 HE 40× **(A)**; CAMTA1 IHC 40× **(B)**; WWTR1::CAMTA1 FISH(+) 40× **(C)**; TFE3 HE 40× **(D)**; TFE3 IHC 40× **(E)**; TFE3 FISH(+) 40× **(F)**;.

IHC showed strong positive expression of vascular endothelial cell markers: ERG 100% (98/98), CD31 98.1% (105/107), CD34 87.7% (93/106), F8 94.3% (50/53). CAMTA1 IHC was positive in 92.2% (83/90) of cases, and TFE3 IHC was positive in 58.0% (51/88) of cases. “FISH showed WWTR1::CAMTA1 fusion was positive in 86.7% (78/90) of cases. The TFE3 break-apart probe was positive (indicating rearrangement) in 13.6% (12/88) of cases (**Figure 1**).

Concordance analysis between IHC and FISH showed substantial agreement for CAMTA1 (Kappa = 0.708, P < 0.001), but poor agreement for TFE3 (Kappa = 0.124, P = 0.055). While there was no statistically significant discordance between CAMTA1 IHC and FISH results, a significant discrepancy was observed for TFE3. Therefore, CAMTA1 IHC serves as a reliable ancillary diagnostic marker, whereas TFE3 status requires confirmation by FISH analysis.

### Histologic atypia is a poor prognosticator in EHE

**Figure 2:**
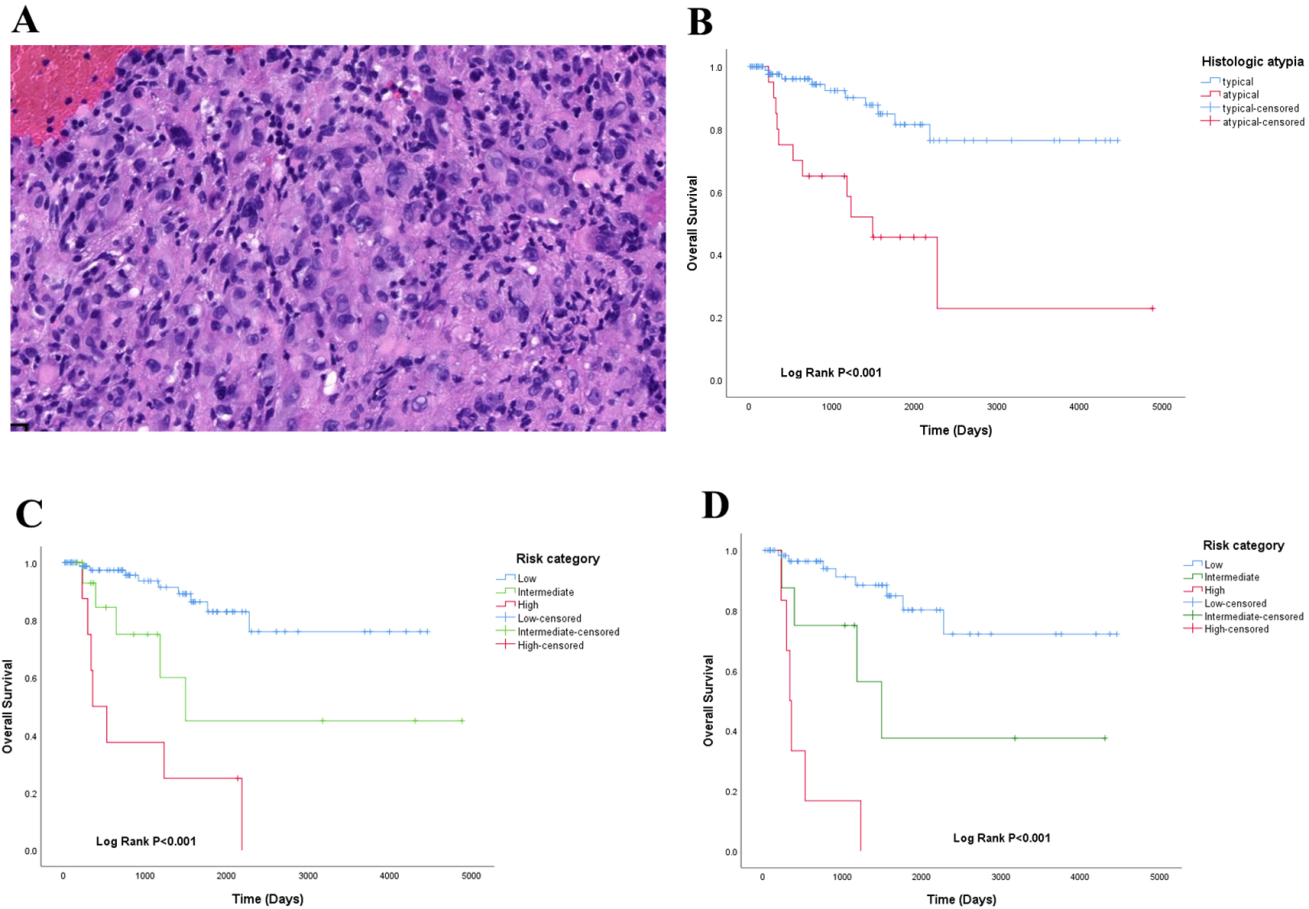
**(A)** histologic atypia in EHE HE 10×;**(B)** overall survival analysis of histologic atypia in EHE; **(C)** a proposed 3-tiered risk model stratified the patients into low-risk, intermedia-risk and high-risk groups; **(D)** risk model of CAMTA1 subtype.

Our retrospective histological review revealed distinct patterns: WWTR1::CAMTA1 EHE tumor cells frequently exhibited cord-like, nested, or infiltrative growth patterns within a hyalinized stromal background. Tumor cells were round to fusiform and often contained characteristic intracytoplasmic vacuoles. In contrast, YAP::TFE3 EHE often demonstrated solid growth with formation of vascular lacunae. Tumor cells in this subtype displayed abundant eosinophilic cytoplasm with ill-defined cell borders (**Figure 1D**). Adopting the criteria established by Takahiro et al. (3), we defined histological atypia as the presence of at least two of three adverse parameters: high mitotic activity (>1 mitosis per 2 mm²), high nuclear grade, and tumor cell necrosis (**Figure 2A**). The presence of histological atypia was significantly associated with poor prognosis (P < 0.001, **Figure 2B**).

### Prognostic features in EHE

The overall 1-year, 3-year, and 5-year cumulative survival rates of EHE were 93.0%, 89.6% and 83.5%, respectively. Then, we assessed the association between clinicopathologic features and EHE prognosis.

**Figure 3:**
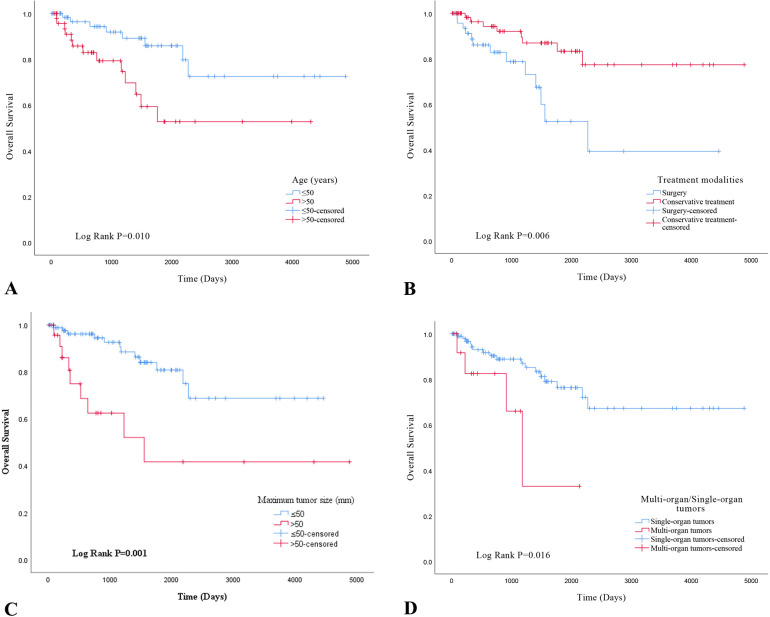
**(A)** overall survival analysis of age in EHE; **(B)** overall survival analysis of treatment modalities in EHE; **(C)** overall survival analysis of maximum tumor size; **(D)** overall survival analysis of multi-organ/single-organ tumors.

Using the Kaplan-Meier method and Log-Rank test, it was determined that EHE with worse OS in the following subgroups: tumor size >50mm (**Figure 3C**), age >50 years old (Figure2-A), multiple organ involvement (**Figure 3D**), conservative treatment (**Figure 3B**) and histologic atypia(**Figure 2B**) (P<0.05).We found that the expression levels of CAMTA1 and TFE3 by IHC (P = 0.152), as well as the presence of the WWTR1-CAMTA1 fusion and TFE3 rearrangement by FISH (P = 0.787), were not statistically correlated with prognosis.

To evaluate the prognostic utility of these parameters, we performed univariate and multivariate Cox regression analyses. Univariate analysis revealed that tumor size >50 mm (P=0.002), age >50 years (P = 0.014), multi-organ involvement (P = 0.024), conservative treatment (P = 0.010), and histological atypia (P < 0.001) were significantly associated with OS. Multivariate analysis confirmed that all five characteristics were independent predictors of OS (**Table 2**; P < 0.05). None of the other selected variables showed a significant association with EHE recurrence.

**Table 2:** Univariable and multivariable COX regression analysis for OS in EHE.

Characteristics	Variable	Univariate analysis	P-value	Multivariate analysis	P-value
		HR (95% CI)		HR (95% CI)	
Gender	Male/female	1.501 (0.631-3.568)	0.358	1.209 (0.467-3.132)	0.696
Age (years)	≤50/>50	3.053 (1.255-7.429)	**0.014**	2.656 (1.024-6.890)	**0.045**
Treatment modalities	Surgery/conservative treatment	0.306 (0.125-0.749)	**0.010**	0.303 (0.107-0.853)	**0.024**
Single/multiple lesions	Single lesions/Multiple lesions	2.631 (0.774-8.948)	0.084	1.867 (0.498-6.994)	0.354
multi-organ/single-organ tumors	multi-organ/single-organ tumors	3.673 (1.187-11.369)	**0.024**	4.441 (1.134-17.399)	**0.032**
Maximum tumor size (mm)	≤50/>50	3.971 (1.660-9.500)	**0.002**	3.419 (1.208-9.679)	**0.021**
Histology	Typical/atypical	4.927 (2.086-11.641)	**<0.001**	2.967 (1.180-7.460)	**0.043**

Bold indicates P < 0.05.

### Risk stratification model of EHE

Based on the previous results, we developed a risk model using five parameters to predict overall survival (**Table 3**). A score (0 or 1) was assigned for each of the following parameters: tumor size (≤50 mm = 0, >50 mm = 1), age (≤50 years = 0, >50 years = 1), treatment modality (surgery = 0, conservative treatment = 1), organ involvement (single-organ = 0, multi-organ = 1), and histology (typical = 0, atypical = 1). The sum of these scores (total score) was used to stratify patients into low-risk (total score 0-1), intermediate-risk (total score 2-3), or high-risk (total score 4-5) groups. Respectively, the survival difference was significant among the 3 groups (P<0.001)(**Figure 2C**). And this prognostic significant using the risk model, was maintained even when the analysis was limited to the CAMTA1 subtype (P<0.001)(**Figure 2D**).

**Table 3:** A proposed system for risk stratification of EHE.

Risk factors	score
Tumor size (mm)	
≤50	0
>50	1
Age (years)	
≤50	0
>50	1
Treatment modalities	
Surgery	0
Conservative treatment	1
Multi-organ/single-organ tumors	
Multi-organ	0
Single-organ tumors	1
Histology	
Typical	0
Atypical*	1
Risk category	Total score
Low	0-1
Intermediate	2-3
High	4-5

*Atypical histology is defined as having at least 2 of the following 3 findings: mitosis >1/2mm2, high nuclear grade, and coagulative tumor necrosis.

## Discussion

In this study, we included 115 EHE patients to investigate the clinicopathologic feature. With the continuous development of research, although most of the classical EHE cases showed low-grade biological manifestations, they had high-grade histopathological characteristics, including aggressive growth patterns, high cell atypia, and high mitotic activity. Consequently, EHE has been classified as 9133/3 in the fifth edition of the WHO Soft Tissue and Bone subvolume, with grading into low-risk and high-risk categories based on the number of mitotic figures and tumor size (4).

Microscopically, epithelioid endothelial tumor cells were nested in the mucous transparent matrix. The intracytoplasmic cavity, containing red blood cells, can be seen. EHE is characterized by epithelioid cells (rich eosinophilic cytoplasm and atypical nucleus), dendritic cells (stellate process), and intermediate cells (features between epithelioid and dendritic cells). Epithelioid cells and dendritic cells may contain cytoplasmic vacuoles with a sig-ring or sac-like appearance (**Figure 1**). Immunohistochemical examination uncovers the presence of endothelial markers, notably CD34, CD31, and ERG, within the tissue samples. Given the acknowledged limitation of CD34 in terms of specificity, it is prudent to adopt a comprehensive approach that incorporates the assessment of CD34 alongside CD31, ERG, F8, and additional pertinent markers for an accurate diagnosis of epithelioid hemangioendothelioma (EHE). In this patient cohort, the positivity rate for CD34 was observed to be 87.7% (93 out of 106 cases), which notably trails behind the 100% positivity of ERG (98 out of 98 cases), 98.1% for CD31 (105 out of 107 cases), and 94.3% for F8 (50 out of 53 cases), emphasizing the importance of a multi-marker evaluation strategy.

According to the fifth edition of the World Health Organization (WHO) classification, the WWTR1::CAMTA1 gene fusion is detected in the vast majority of epithelioid hemangioendothelioma (EHE) cases, specifically in over 90% and in the prior study by Doyle et al (1), CAMTA1 positivity was reported at 86%. This finding is consistent with our results, which demonstrate CAMTA1 immunohistochemical (IHC) positivity in 92.2% (83 of 90) of analyzed cases and TFE3 IHC positivity in 58.0% (51 of 88) of cases.

Furthermore, FISH analysis revealed a WWTR1::CAMTA1 fusion positivity rate of 86.7% (78/90), underscoring the prevalence of this genetic aberration. Conversely, the frequency of TFE3 rearrangements detected by FISH was relatively low at 13.6% (12/88), highlighting the differential genetic underpinnings associated with these markers in EHE.

The molecular basis for this phenomenon stems from the structure of the WWTR1::CAMTA1 fusion protein, which combines the N-terminal portion of WWTR1 with the C-terminal region of CAMTA1. Notably, while CAMTA1 constitutes the major component of the fusion protein, the N-terminal fragment of WWTR1 retains its TEAD-binding motif, enabling it to function as a potent co-activator that triggers the activation of transcriptional programs. This fusion results in constitutive activation of WWTR1, which promotes abnormal cell proliferation, inhibits tumor cell death via autophagy, and ultimately drives tumor growth and progression (5).

Concurrently, the WWTR1::CAMTA1 fusion protein retains the N-terminal domain of WWTR1 (containing the TEAD-binding domain) and the C-terminal domain of CAMTA1 (containing the nuclear localization signal and the transcriptional activation domain). This structural configuration confers constitutive nuclear trafficking capability to the fusion protein, rendering it unresponsive to physiological regulatory mechanisms. This forced nuclear localization accounts for the distinct and homogeneous nuclear positivity pattern observed in CAMTA1 immunohistochemistry (IHC), which exhibits strong concordance with the gene break-apart signals detected by fluorescence *in situ* hybridization (FISH) (6).

In contrast to the singularity of the WWTR1::CAMTA1 fusion, EHE with TFE3 rearrangements predominantly features YAP1::TFE3 fusions, although other rare partner genes exist. TFE3 demonstrates highly heterogeneous fusion partners. Reported partners in the literature include YAP1 and WWTR1, among others (6, 7).

The YAP1::TFE3 fusion, representing the canonical subtype, results in the retention of TFE3’s C-terminal DNA-binding domain while its N-terminus is replaced by the transcriptional activation domain of YAP1 (7). This structural alteration may impact the half-life of the TFE3 protein, rendering it more susceptible to degradation during tissue processing. The complexity is further increased by the novel WWTR1::TFE3 fusion variant recently reported by Li et al. (6). This fusion retains TFE3’s bHLH and leucine zipper domains; however, a critical serine residue mutation within the WWTR1 moiety abrogates its binding capacity to TEAD, potentially indirectly affecting protein stability.

Furthermore, research endeavors have illuminated the role of connective tissue growth factor (CTGF), a transcriptional target of the WWTR1::CAMTA1 fusion protein, in fostering carcinogenesis through the overactivation of the MAPK signaling pathway. This revelation underscores the potential of MEK inhibitor trametinib, which targets the CTGF-MAPK axis, as a promising targeted therapeutic approach for EHE patients who harbor the WWTR1::CAMTA1 fusion. By disrupting this oncogenic signaling cascade, trametinib holds the promise of inhibiting tumor growth and progression in this specific subset of EHE patients (8, 9).

Distinct from typical oncogenes, YAP1 and WWTR1 do not independently initiate spontaneous cancer development, though they are indispensable for tumor progression. Activation of the Hippo pathway triggers phosphorylation and subsequent sequestration of YAP1 and WWTR1 within the cytoplasm, thereby facilitating the activation of alternative oncogenic signaling cascades, including those mediated by EPCR, Wnt, NOTCH, and RAS. This intricate interplay underscores the complexity of the molecular mechanisms underlying EHE and highlights the importance of understanding the role of gene fusions and signaling pathway crosstalk in tumor biology (10). Given the robust nuclear localization signal of CAMTA1, the nuclear localization of the WWTR1::CAMTA1 fusion protein, coupled with its ability to recruit chromatin remodeling factors, facilitates the induction of carcinogenic transcriptional programs (11).

In NCC cohort, tumor size and histological atypia were significantly associated with shorter overall survival (OS), corroborating findings from prior studies (12, 13). Regarding tumor size determination, which is often problematic in EHE due to multifocality and multiorgan involvement, we combined computed tomography (CT) or other imaging modalities with histological evaluation. For patients with multifocal nodules, the maximum diameter of the largest nodule was recorded using imaging (CT or MRI). Imaging (CT or MRI) was performed in nearly all patients to measure nodules *in vivo*.

Currently, surgical intervention remains the primary treatment approach for EHE, although a subset of patients are not candidates for radical resection. Our investigation revealed that patients undergoing radical resection had a more favorable prognosis than those receiving conservative management. Furthermore, we found that, beyond tumor size and histological atypia, age and multiorgan involvement were also significantly associated with shorter OS. As multivariable analysis identified these as independent prognostic factors, we developed a novel risk model for the first time using these five parameters. This risk model remained valid for the CAMTA1 subtype but not for the TFE3 subtype, likely attributable to the limited number of TFE3-subtype cases included. This simple three-tiered risk stratification system significantly discriminated EHE prognoses, with estimated 5-year OS rates of 91.2%, 68.8%, and 12.5% for the low-, intermediate-, and high-risk groups, respectively.

In summary, our study definitively confirms that tumor size exceeding a specific millimeter threshold, age over 50 years, surgical intervention (or lack thereof), histological atypia, and multiorgan involvement in EHE are all significantly associated with shorter OS in both univariate and multivariate analyses. Utilizing these five parameters, we devised a three-tiered risk model that effectively stratified patients into three groups with significantly distinct OS rates. While prior studies have primarily focused on tumor size and histological atypia, our model incorporates additional clinical factors (age, treatment modality, multiorgan involvement). This broader scope enables a preliminary prognostic assessment for EHE patients where histology is indeterminate and is particularly applicable for pre-operative risk evaluation, encompassing tumor burden (size, multiorgan status), age, and treatment intervention. Notably, the intermediate-risk group in our current model exhibited a significantly lower survival rate (68.8% *vs*. 81.8% reported elsewhere), suggesting its enhanced ability to identify patients with potential adverse outcomes earlier. Particularly for biopsy specimens, this model may help mitigate, to some extent, the inherent subjectivity unavoidable in histological assessment.

In conclusion, our study has conclusively demonstrated that tumor size exceeding a specific millimeter threshold, age over 50 years, surgical intervention, histological atypia, and multi-organ involvement in EHE are significantly correlated with a shorter overall survival (OS) in both univariate and multivariate analyses. Leveraging these five parameters, we have devised a three-tiered risk model that effectively stratifies patients into three distinct groups with significantly different OS rates. A comparative analysis between fluorescence *in situ* hybridization (FISH) and immunohistochemistry (IHC) revealed good concordance for detecting CAMTA1 alterations, whereas the consistency for TFE3 was suboptimal. Consequently, we advocate the use of IHC for CAMTA1 diagnosis and recommend FISH for TFE3 assessment. To propel advancements in EHE diagnosis and treatment, a profound understanding of its molecular biology and genetic underpinnings is paramount. This knowledge will pave the way for more targeted and effective therapeutic strategies tailored to the unique characteristics of this rare malignancy.

## Data Availability

The raw data supporting the conclusions of this article will be made available by the authors, without undue reservation.
